# Supplementary material for: Unveiling the Bioactive Potential of the Invasive Jellyfish Phyllorhiza punctata Through Integrative Transcriptomic and Proteomic Analyses
Source: Biomolecules. 2025 Aug 4;15(8):1121. doi: 10.3390/biom15081121 (PMC12383608; doi:10.3390/biom15081121)
Supplement: Supplementary file 1 [file biomolecules-15-01121-s001.zip › Suplementary Material/FileS3/PEPFOLD4/XP_065051566_5519/clusters.html]

 PEP-FOLD clusters


Download archive of all models  here   

|  |  |  |
| --- | --- | --- |
| # | file | sOPEP |
| # cluster 1 - Size: 7 |  |  |
|  | XP\_065051566\_5519-model1 | -8.02867 |
|  | XP\_065051566\_5519-model1.1 | -8.02867 |
|  | XP\_065051566\_5519-model1.2 | -8.02867 |
|  | XP\_065051566\_5519-model1.3 | -8.02867 |
|  | XP\_065051566\_5519-model1.4 | -7.90959 |
|  | XP\_065051566\_5519-model1.5 | -7.90959 |
|  | XP\_065051566\_5519-model1.6 | -7.77161 |
| # cluster 2 - Size: 93 |  |  |
|  | XP\_065051566\_5519-model2 | -7.96378 |
|  | XP\_065051566\_5519-model2.1 | -7.96378 |
|  | XP\_065051566\_5519-model2.2 | -7.96378 |
|  | XP\_065051566\_5519-model2.3 | -7.96378 |
|  | XP\_065051566\_5519-model2.4 | -7.96378 |
|  | XP\_065051566\_5519-model2.5 | -7.96378 |
|  | XP\_065051566\_5519-model2.6 | -7.96378 |
|  | XP\_065051566\_5519-model2.7 | -7.96378 |
|  | XP\_065051566\_5519-model2.8 | -7.96378 |
|  | XP\_065051566\_5519-model2.9 | -7.96378 |
|  | XP\_065051566\_5519-model2.10 | -7.96378 |
|  | XP\_065051566\_5519-model2.11 | -7.96378 |
|  | XP\_065051566\_5519-model2.12 | -7.96378 |
|  | XP\_065051566\_5519-model2.13 | -7.96378 |
|  | XP\_065051566\_5519-model2.14 | -7.96378 |
|  | XP\_065051566\_5519-model2.15 | -7.96378 |
|  | XP\_065051566\_5519-model2.16 | -7.96378 |
|  | XP\_065051566\_5519-model2.17 | -7.96378 |
|  | XP\_065051566\_5519-model2.18 | -7.96378 |
|  | XP\_065051566\_5519-model2.19 | -7.96378 |
|  | XP\_065051566\_5519-model2.20 | -7.96378 |
|  | XP\_065051566\_5519-model2.21 | -7.96378 |
|  | XP\_065051566\_5519-model2.22 | -7.96378 |
|  | XP\_065051566\_5519-model2.23 | -7.96378 |
|  | XP\_065051566\_5519-model2.24 | -7.96378 |
|  | XP\_065051566\_5519-model2.25 | -7.96378 |
|  | XP\_065051566\_5519-model2.26 | -7.96378 |
|  | XP\_065051566\_5519-model2.27 | -7.96378 |
|  | XP\_065051566\_5519-model2.28 | -7.96378 |
|  | XP\_065051566\_5519-model2.29 | -7.96378 |
|  | XP\_065051566\_5519-model2.30 | -7.96378 |
|  | XP\_065051566\_5519-model2.31 | -7.96378 |
|  | XP\_065051566\_5519-model2.32 | -7.96378 |
|  | XP\_065051566\_5519-model2.33 | -7.96378 |
|  | XP\_065051566\_5519-model2.34 | -7.96378 |
|  | XP\_065051566\_5519-model2.35 | -7.96378 |
|  | XP\_065051566\_5519-model2.36 | -7.96378 |
|  | XP\_065051566\_5519-model2.37 | -7.96378 |
|  | XP\_065051566\_5519-model2.38 | -7.96378 |
|  | XP\_065051566\_5519-model2.39 | -7.96378 |
|  | XP\_065051566\_5519-model2.40 | -7.96378 |
|  | XP\_065051566\_5519-model2.41 | -7.96378 |
|  | XP\_065051566\_5519-model2.42 | -7.96378 |
|  | XP\_065051566\_5519-model2.43 | -7.96378 |
|  | XP\_065051566\_5519-model2.44 | -7.96378 |
|  | XP\_065051566\_5519-model2.45 | -7.96378 |
|  | XP\_065051566\_5519-model2.46 | -7.96378 |
|  | XP\_065051566\_5519-model2.47 | -7.96378 |
|  | XP\_065051566\_5519-model2.48 | -7.96378 |
|  | XP\_065051566\_5519-model2.49 | -7.96378 |
|  | XP\_065051566\_5519-model2.50 | -7.96378 |
|  | XP\_065051566\_5519-model2.51 | -7.96378 |
|  | XP\_065051566\_5519-model2.52 | -7.96378 |
|  | XP\_065051566\_5519-model2.53 | -7.96378 |
|  | XP\_065051566\_5519-model2.54 | -7.96378 |
|  | XP\_065051566\_5519-model2.55 | -7.96378 |
|  | XP\_065051566\_5519-model2.56 | -7.96378 |
|  | XP\_065051566\_5519-model2.57 | -7.96378 |
|  | XP\_065051566\_5519-model2.58 | -7.96378 |
|  | XP\_065051566\_5519-model2.59 | -7.96378 |
|  | XP\_065051566\_5519-model2.60 | -7.96378 |
|  | XP\_065051566\_5519-model2.61 | -7.96378 |
|  | XP\_065051566\_5519-model2.62 | -7.96378 |
|  | XP\_065051566\_5519-model2.63 | -7.96378 |
|  | XP\_065051566\_5519-model2.64 | -7.96378 |
|  | XP\_065051566\_5519-model2.65 | -7.96378 |
|  | XP\_065051566\_5519-model2.66 | -7.96378 |
|  | XP\_065051566\_5519-model2.67 | -7.96378 |
|  | XP\_065051566\_5519-model2.68 | -7.96378 |
|  | XP\_065051566\_5519-model2.69 | -7.96378 |
|  | XP\_065051566\_5519-model2.70 | -7.96378 |
|  | XP\_065051566\_5519-model2.71 | -7.96378 |
|  | XP\_065051566\_5519-model2.72 | -7.96378 |
|  | XP\_065051566\_5519-model2.73 | -7.96378 |
|  | XP\_065051566\_5519-model2.74 | -7.96378 |
|  | XP\_065051566\_5519-model2.75 | -7.96378 |
|  | XP\_065051566\_5519-model2.76 | -7.96378 |
|  | XP\_065051566\_5519-model2.77 | -7.96378 |
|  | XP\_065051566\_5519-model2.78 | -7.96378 |
|  | XP\_065051566\_5519-model2.79 | -7.96378 |
|  | XP\_065051566\_5519-model2.80 | -7.96378 |
|  | XP\_065051566\_5519-model2.81 | -7.96378 |
|  | XP\_065051566\_5519-model2.82 | -7.96378 |
|  | XP\_065051566\_5519-model2.83 | -7.96378 |
|  | XP\_065051566\_5519-model2.84 | -7.96378 |
|  | XP\_065051566\_5519-model2.85 | -7.96378 |
|  | XP\_065051566\_5519-model2.86 | -7.96378 |
|  | XP\_065051566\_5519-model2.87 | -7.96378 |
|  | XP\_065051566\_5519-model2.88 | -7.96378 |
|  | XP\_065051566\_5519-model2.89 | -7.96378 |
|  | XP\_065051566\_5519-model2.90 | -7.96378 |
|  | XP\_065051566\_5519-model2.91 | -7.87684 |
|  | XP\_065051566\_5519-model2.92 | -7.87684 |
|  |  |  |
